# Anxiety disorders and the gut microbiota: a bibliometric and visual analysis

**DOI:** 10.3389/fpsyt.2024.1517508

**Published:** 2025-01-20

**Authors:** Linli Guo, Qin Ding, Qing Li, Danping Zheng, Linglin Guo, Xiaotao Cao, Qianqian Mou

**Affiliations:** ^1^ Department of Clinical Trial Center, West China Hospital, Sichuan University, Chengdu, Sichuan, China; ^2^ Department of West China School of Nursing, Sichuan University, Chengdu, Sichuan, China; ^3^ Department of Mental Health Center, West China Hospital, Sichuan University, Chengdu, Sichuan, China; ^4^ Department of Outpatient Department, West China Hospital, Sichuan University, Chengdu, Sichuan, China; ^5^ Department of Neurosurgery, West China Hospital, Sichuan University, Chengdu, Sichuan, China

**Keywords:** anxiety disorder, gut microbe, gut-brain axis, bibliometrics, immunity

## Abstract

**Introduction:**

Anxiety disorder is a common mental illness, yet its specific mechanisms remain unclear. Recent research has revealed a connection between gut microbiota and anxiety disorders. This study aims to assess the current global research landscape, highlight current topics of interest, and explore future research directions in the field of anxiety disorders and gut microbiota.

**Methods:**

We extracted research review articles related to anxiety and gut microbiota from the Web of Science, covering the period from 2004 to 2023. We used VOSviewer 1.6.18.0, Scimago Graphica, and CiteSpace 6.2. R2 to visualize the contributions of countries, institutions, journals, authors, citations, and keywords in this field.

**Result:**

A total of 1198 articles were included in this bibliometric analysis. Over the past two decades, both publications and citations have shown a steady increase. China, the United States, and Canada were the top three countries in terms of publication output. John Cryan from University College Cork had the highest number of publications and citation impact in this area of research. The journal Nutrients had the highest number of publications, while Brain Behavior and Immunity had the most citations. Key research themes in recent years have included anxiety, gut microbiota, depression, stress, gut-brain axis, and probiotics, all of which are likely to be important future research directions.

**Conclusion:**

This analysis has key research areas and emerging trends, including risk factors, stressors, inflammatory responses, the gut-brain axis, and probiotics. These insights can guide researchers towards a more comprehensive understanding of recent advancements in this field, help shape future research directions and facilitate the identification of new therapeutic targets for anxiety disorder, ultimately improving clinical outcomes.

## 1 Introduction

Anxiety disorders are among the most common mental illnesses worldwide (1). They are primarily characterized by excessive worry and nervousness, often accompanied by dry mouth and sweating and are more common in women (2). Globally, the current global prevalence of anxiety disorders is estimated at 7.3% (ranging between 4.8 and 10.9%) (3). Since the onset of the COVID-19 pandemic, the prevalence may have increased more than threefold (4). Additionally, anxiety disorders often do not occur in isolation, they frequently co-occur with other psychiatric or somatic conditions. For instance, 16.7% of patients with epilepsy and 11% of those with chronic obstructive pulmonary disease (COPD) are diagnosed with anxiety disorder (5). Despite its prevalence, the exact pathogenesis of anxiety disorders remains unclear (6). However, emerging evidence suggests a potential link between the occurrence of anxiety disorders and the regulatory function of gut microbiota (7).

The human microbiota, a complex and diverse ecosystem of trillions of bacteria, viruses, fungi, and other microorganisms, is predominately located in the intestines (8). This microbial community varies between individuals and is influenced by factors such as birth method, genetics, environment, stress, infections, and antibiotic use (9). The bidirectional communication between the gastrointestinal tract and the brain, often referred to as “gut-brain axis”, is thought to play a crucial role in this relationship (10). Changes in the composition of the gut microbiota can impact intestinal permeability, mediate inflammation and immune responses, and produce toxic metabolites (11). These toxic metabolites may cross the blood-brain barrier (BBB), triggering inflammation in the brain and affecting neural pathways (11). The gut microbiota also regulates the hypothalamic-pituitary-adrenal (HPA) axis, as well as neurotransmitters such as norepinephrine, dopamine, and Gamma-aminobutyric acid (GABA), by affecting their synthesis and metabolism (7, 9). Therefore, gut microbiota imbalance may provide new insights into the pathogenesis of anxiety disorders.

Bibliometric analysis is a widely used research tool (12) that employs both qualitative and quantitative methods to evaluate scientific literature. Analyzing published journals helps identify research hotspots and trends, providing valuable guidance for future studies (13). While some bibliometric studies have investigated the relationship between gut microbiota, depression, and the gut-brain axis, no such analysis has yet been conducted on anxiety disorders and gut microbiota. This study aims to fill that gap by performing a visual and bibliometric analysis of research on the relationship between gut microbiota and anxiety disorders, based on a wide range of bibliometric indicators. Our goal is to summarize current findings, highlight emerging trends, and offer guidance for future research in the field.

## 2 Survey methodology

### 2.1 Data collection

To ensure data accuracy and representativeness, we downloaded relevant data on anxiety disorders and gut microbiota from the Web of Science Core Collection (WoSCC) on March 30, 2024. The research query used was TS= (Anxiety disorder) AND [(Intestinal flora) OR (gut microbiota)], covering publications from January 1 2004 to December 31, 2023. To reduce bias, we selected only research and review articles written in English, ultimately identifying 1198 articles meeting the inclusion criteria.

### 2.2 Data analysis

For the bibliometric analysis, we used VOSviewer 1.6.18.0, a versatile software for constructing and visualizing bibliometric maps (14). CiteSpace 6.2.R2 was employed to analyze emerging trends within the field by analyzing keywords, research institutions, and other data. In addition, Scimago Graphica was used to visualize the geographic distribution of the publications. These tools were applied to generate visual analysis of countries, institutions, journals, years, authors and keywords related to the selected literature.

## 3 Results

### 3.1 Annual output and citation trends

**Figure 1:**
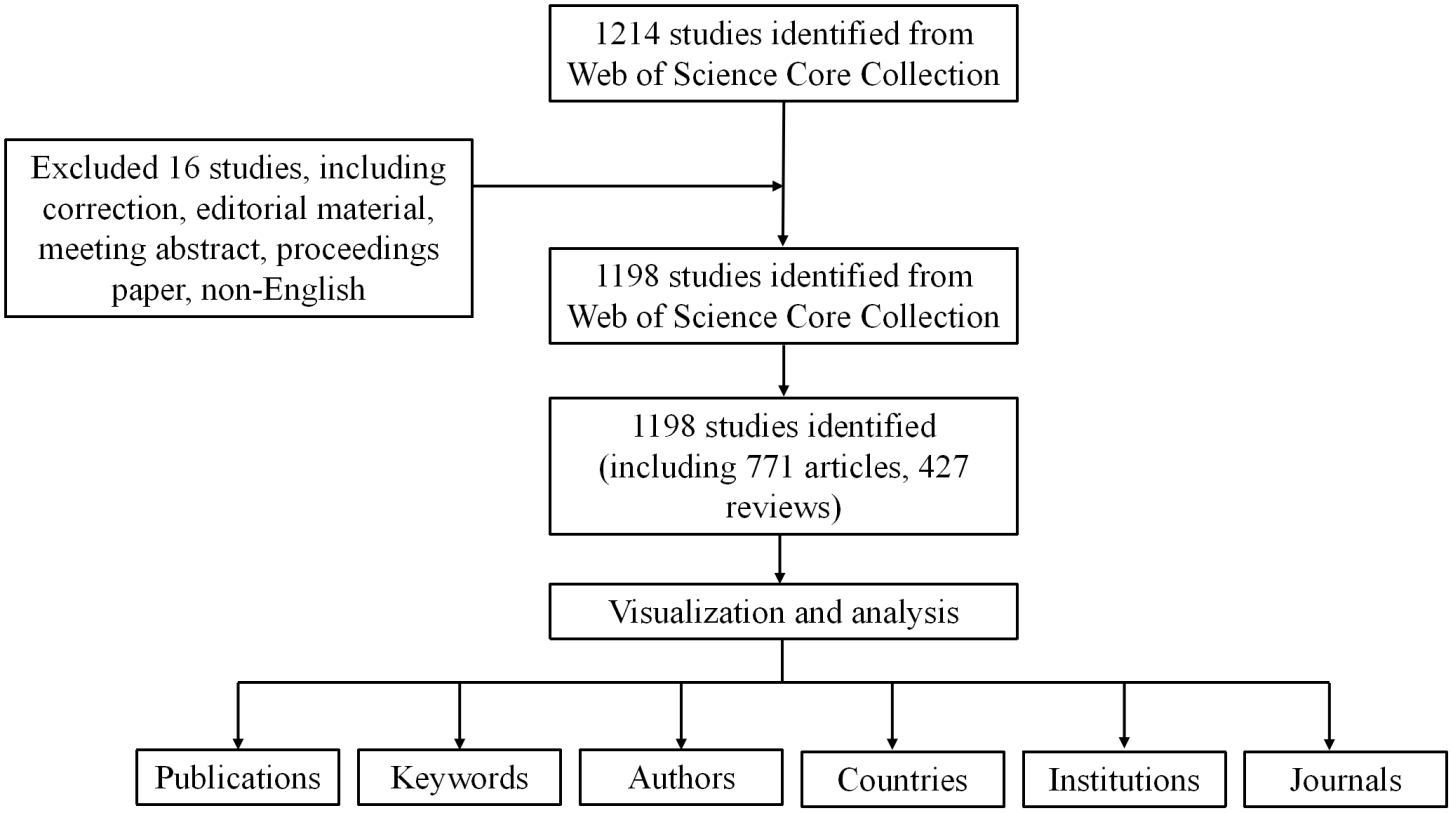
Flow chart.

**Figure 2:**
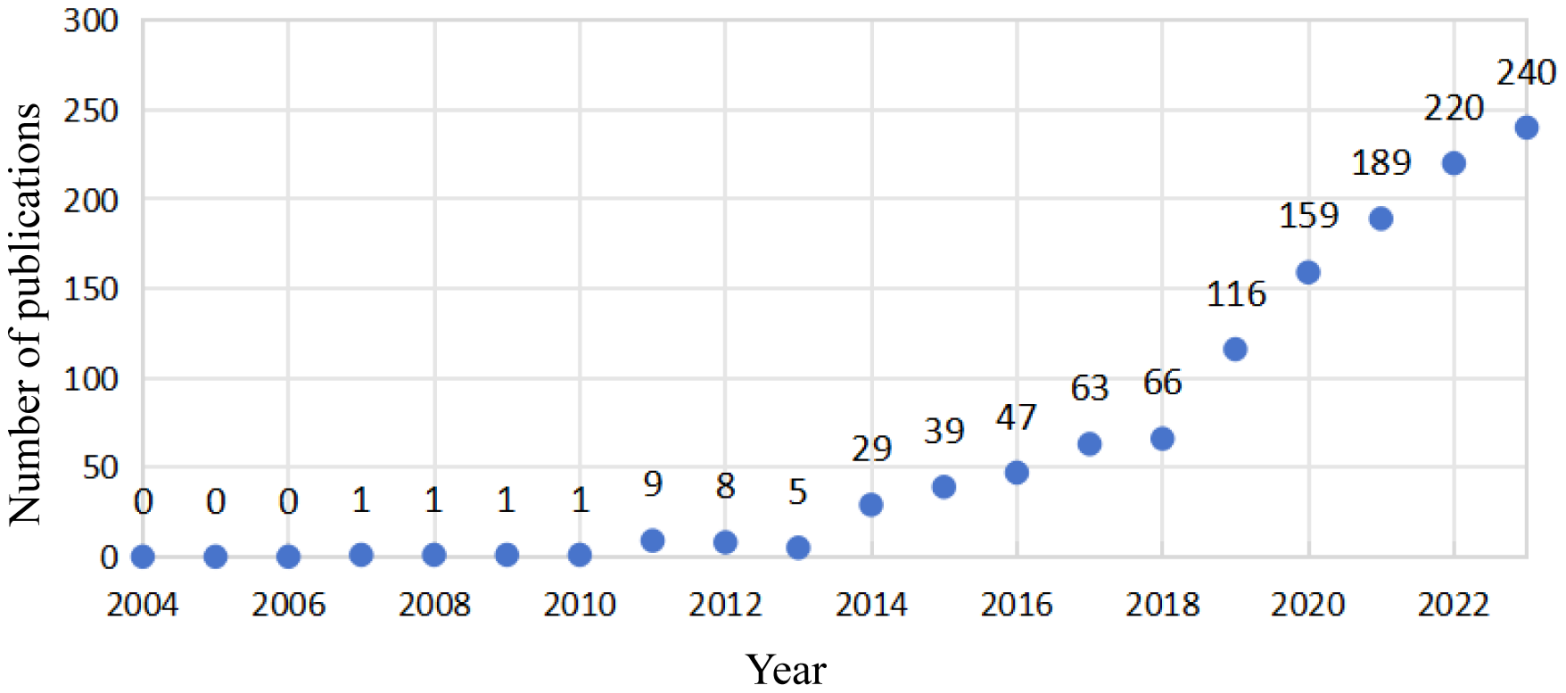
Annual publications and citations on anxiety disorders and gut microbiota from 2004 to 2023.

As shown in **Figure 1**, a total of 1198 studies met the search criteria. As shown in **Figure 2**, no relevant articles were published between 2004 and 2006, with the first article published in 2007. Slow growth followed from 2011, reaching a stable period between 2015 and 2018, during which an average of 53 articles were published per year. Starting in 2019, the field experienced a surge in publications, increasing from 66 to 116 articles annually, indicating growing interest among researchers. In the last five years alone, 924 articles were published, accounting for 77.1% of the total, indicating that research in this field has gained significant momentum and maturity.

### 3.2 Most productive countries and institutions

**Figure 3:**
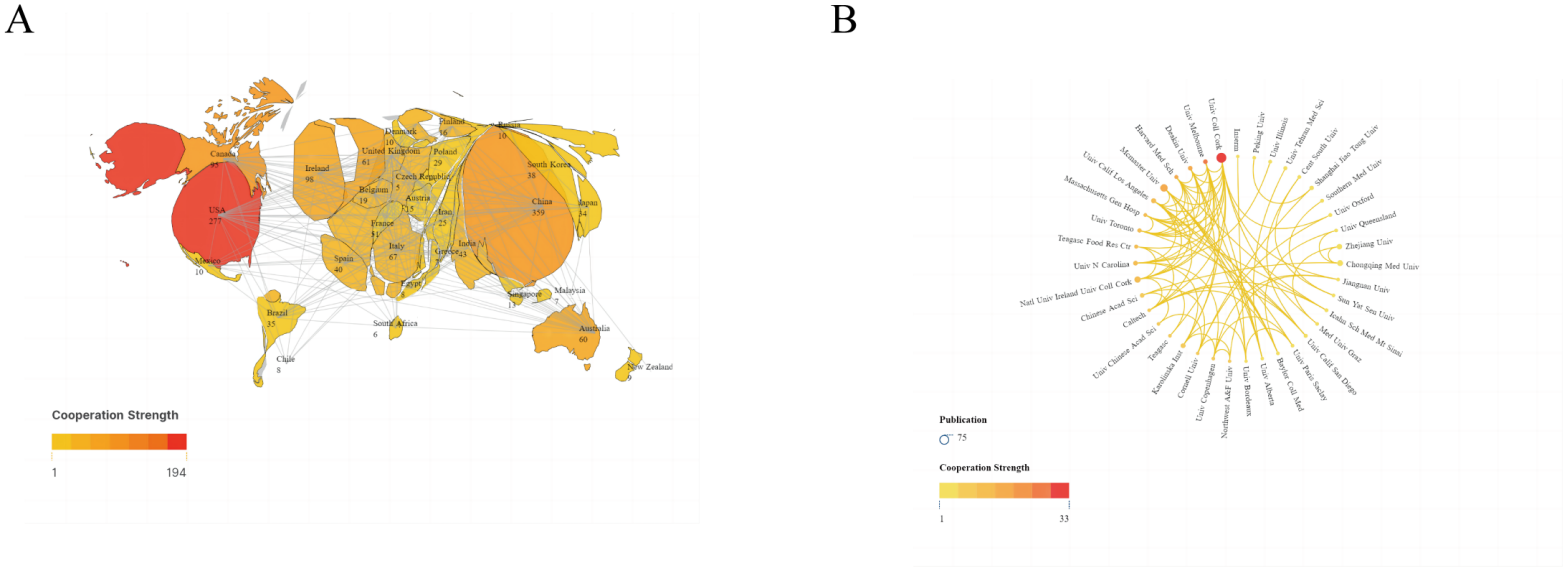
**(A)** Publications and cooperation networks of countries. **(B)** Publications and cooperation networks of institutions. Denser and deeper links between two circles indicate closer collaboration.

The 1,198 studies identified included 771 articles and 427 reviews. We performed a visual analysis of the 41 countries with five or more publications using the VOSviewer software. As shown in **Figure 3A**, the map highlights publication output from various countries and international collaborations, with deeper colors indicating stronger cooperation between countries. China had the highest number of publications (359, 29.97%), followed by the United States (277, 23.12%), Ireland (98, 8.18%), Canada (95, 7.9%), and Italy (67, 5.59%). The top five countries in terms of collaborative intensity were the United States (194), China (110), Canada (84), Ireland (76), and the United Kingdom (72) (**Table 1**). These countries not only produced the most publications but also maintained strong international cooperation with other countries. In terms of citation impact, Ireland led with 21573 citations, indicating high international recognition in the field of anxiety and gut microbiota research.

**Table 1:** Counts, citations, and total link strengths (TLS) of publications of different countries and institutions.

Rank	Country	Count Number	Citations	TLS	Institution (Country)	Count Number	Citations	TLS
1	China	359	10535	110	University College Cork	66	10810	33
2	USA	277	17053	194	McMaster University	37	9015	15
3	Ireland	98	21573	76	National University of Ireland	26	10543	11
4	Canada	95	12359	84	Chongqing Medical University	23	2113	3
5	Italy	67	2389	50	Zhejiang University	20	1708	2
6	Australia	60	4120	67	University of California, Los Angeles	17	2975	14
7	England	54	2476	72	Karolinska Institute	16	746	9
8	France	51	3019	37	Carolina University	15	611	11
9	India	43	703	29	Chinese Acad Sci Deakin University University of Melbourne	14 14 14	904 770 935	10 19 24
10	Spain	40	1646	38	Shanghai Jiao Tong University	13	395	2

We also conducted a visual analysis of 39 institutions that had published more than eight publications, As shown in **Figure 3B**, the top five institutions were University College Cork (66 articles), McMaster University (37 articles), National University of Ireland (26 articles), Chongqing Med University (23 articles), and Zhejiang University (20 articles) (**Table 1**). These institutions hailing from Ireland, Canada, and China, show the closest collaborations with University College Cork, highlighting its prominent international influence in anxiety disorder and gut microbiota research.

### 3.3 Co-authors and co-cited authors

**Figure 4:**
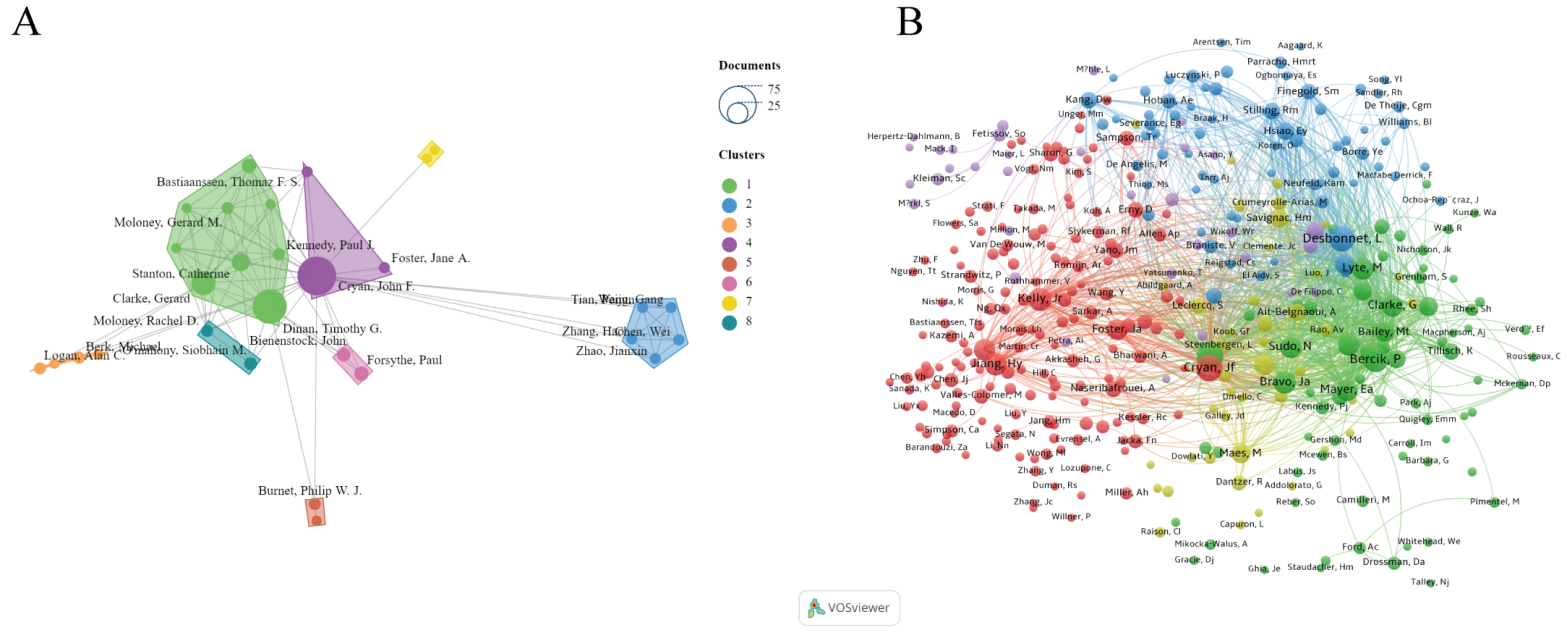
**(A)** Network visualization map of the authors. **(B)** Network visualization map of the co-cited authors.

As shown in **Figure 4**, the size of nodes represents the number of papers published by each author. The most prolific author is John Cryan from University College Cork, Ireland, with 73 articles and 16,023 citations. He is followed by Timothy Dinan (57 articles), Gerard Clarke (28 articles), Catherine Stanton (16 articles), and Thomaz Bastiaanssen (10 articles) (**Table 2**). These authors, mainly from Ireland, are the top five researchers in the last five years and pioneers in thematic research on anxiety disorders and gut microbiota. The figure also illustrates connections between authors, with more connections indicating closer collaborations. John Cryan has the highest collaborations, followed by Timothy Dinan, Gerard Clarke, Catherine Stanton, and Thomaz Bastiaanssen. This suggests the need for more international cooperation to advance research in this area.

**Table 2:** Top 10 co-authors and co-cited authors related to anxiety disorder and gut microbiota.

Rank	Co-author	Count Number	Citations	TLS	Co-cited author	Citations	TLS
1	Cryan, JF	73	16023	169	Cryan, JF	625	23660
2	Dinan, TG	57	14259	149	Dinan, TG	615	28265
3	Clarke, G	28	6292	99	Desbonnet, L	541	26975
4	Stanton, C	16	3397	54	Bercik, P	537	24825
5	Thomaz F. S. Bastiaanssen Forsythe, P	10 10	386 3481	41 11	Kelly, JR	428	18202
6	Bienenstock, John	9	3673	15	Bravo, JA	406	16530
7	O'mahony, SM	8	877	15	Jiang, HY	386	14080
8	Berk, Michael Burnet, Philip WJ Kennedy, PJ Logan, AC Moloney, GM Moloney, RD	7 7 7 7 7 7	509 635 2203 789 355 1964	9 5 29 1 34 24	Omahony, SM	367	19960
9	Chen, Wei Foster, JA Rea, K Tian, Peijun Wang, Gang Zhang, Hao Zhao, Jianxin	6 6 6 6 6 6 6	244 922 376 244 244 254 244	24 2 14 24 24 21 24	Lyte, M	364	20070
10	Anthony, Aaniel C. Berding,K Hoban, AE Hsiao, EY Jacka, F Jacka, FN. Mazmanian, SK Stilling, RM.	5 5 5 5 5 5 5 5	167 96 1288 2747 208 331 4202 1038	3 21 24 1 5 5 2 23	Clarke, G	363	19386

Co-cited authors are those cited together in two or more articles. A total of 376 authors were co-cited, with 69 authors co-cited more than 100 times. The top five co-cited authors were John Cryan (625 times), Timothy Dinan (615 times), Lieve Desbonnet (541 times), Premsyl Bercik (537 times), and John R. Kelly (428 times).

### 3.4 Journals and co-cited journals

**Figure 5:**
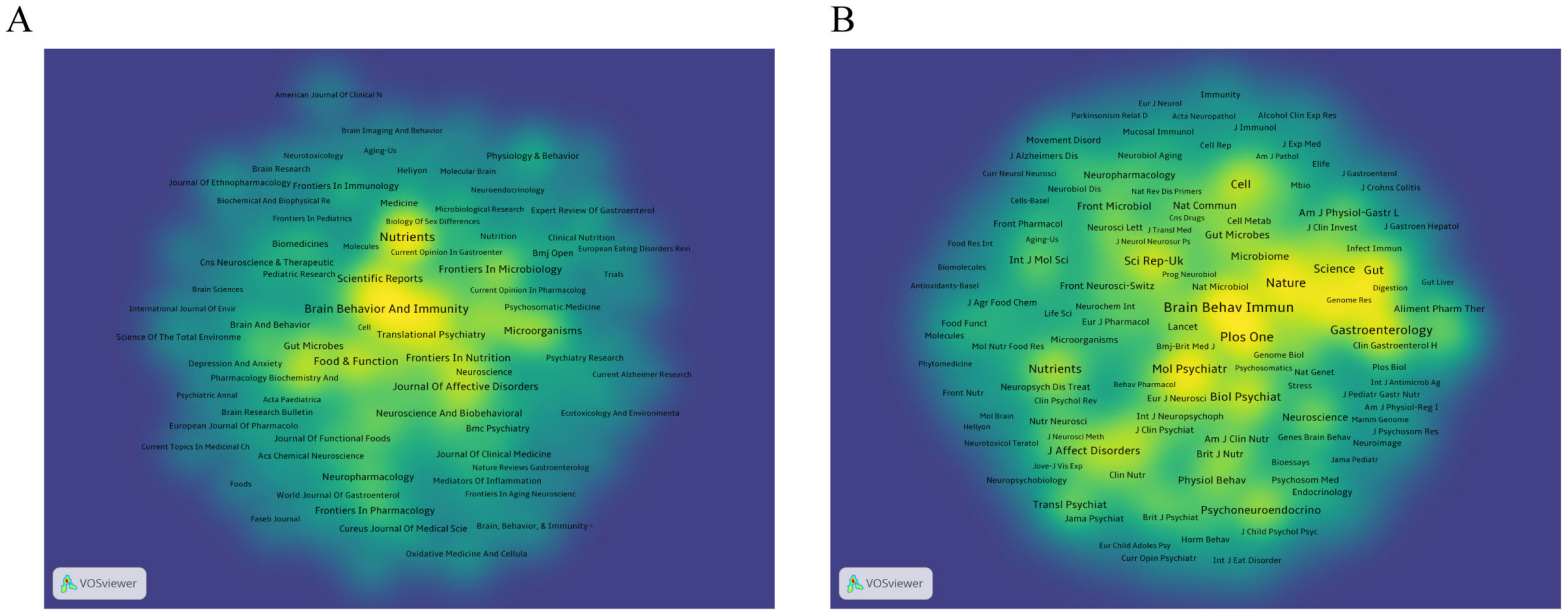
**(A)** Density visualization of journals. **(B)** Density visualization of co-cited journals. The citing journals are on the left, and the cited journals are on the right. Colorful paths represent their relationship.

We used VOSviewer software to visualize 188 journals that had published more than two articles on anxiety disorders and gut microbiome research. As shown in **Figure 5A**, The top five journals were Nutrients, Brain, Behavior, and Immunity (Brain Behav Immun), Frontiers in Psychiatry, Food & Function, and Frontiers in Neuroscience (**Table 3**). These journals are mainly published in Switzerland, the United States, and the United Kingdom, reflecting a strong focus on this field in developed countries. This indicates that developed countries may be leading in research on gut microbiota and anxiety disorders compared to other countries around the world.

As shown in **Figure 5B**, The top five most cited journals were Brain Behav Immun, PLOS ONE (Plos One), Gastroenterology, Proceedings of the National Academy of Sciences (PNAS), and Nature (**Table 3**), all of which were cited more than 1900 times.

**Table 3:** Top 10 journals and co-cited journals related to anxiety disorder and gut microbiota.

Rank	Journal	Count Number	IF (2023)	JCR	Journal	Citation	IF (2022)	JCR
1	Nutrients	45	4.8	Q2	Brain Behav Immun	2790	8.8	Q2
2	Brain Behavior And Immunity	37	8.8	Q2	Plos One	2382	2.9	Q3
3	Frontiers In Psychiatry	22	3.2	Q3	Gastroenterology	2145	25.7	Q1
4	Food & Function Frontiers In Neuroscience	21 21	5.1 3.2	Q1 Q3	P Natl Acad Sci Usa	2124	9.4	Q1
5	International Journal Of Molecular Sciences Scientific Reports	20 20	4.9 3.8	Q2 Q2	Nature	1954	50.5	Q1
6	Frontiers In Microbiology Journal Of Affective Disorders	17 17	4.0 .9	Q2 Q2	Neurogastroent Motil	1828	3.5	Q3
7	Frontiers In Nutrition Progress In Neuro-Psychopharmacology & Biological Psychiatry	16 16	4.0 5.3	Q2 Q2	Gut	1591	23	Q1
8	Frontiers In Cellular And Infection Microbiology Microorganisms Plos One	15 15 15	4.6 4.1 2.9	Q2 Q2 Q3	Sci Rep-Uk	1581	3.8	Q2
9	Gut Microbes Translational Psychiatry	13 13	12.2 5.8	Q1 Q1	Mol Psychiatr	1555	9.6	Q1
10	Behavioural Brain Research	12	2.6	Q3	Cell	1462	45.5	Q1

### 3.5 Keywords

**Figure 6:**
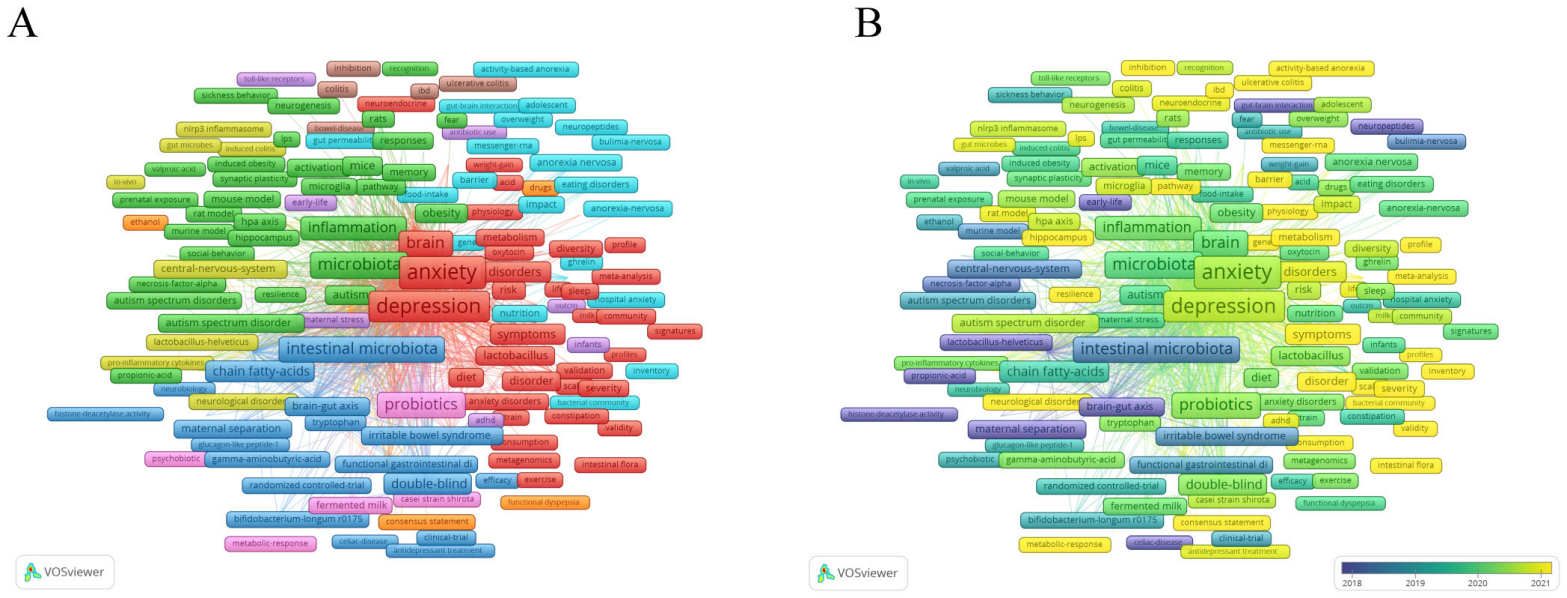
**(A)** Overlay visualization with relevant keywords anxiety disorder and gut microbiota. **(B)** Temporal changes in keywords related to anxiety and gut.

Keyword co-occurrence analysis helps to identify research hotspots and development trends in this research field. We extracted 4224 keywords and visually analyzed 508 that appeared more than five times using VOSviewer. As shown in **Figure 6A**, the top 10 most frequently occurring keywords were anxiety (559), gut microbiota (518), depression (470), microbiota (267), stress (244), gut-brain axis (237), brain (222), intestinal microbiota (212), probiotics (209), and anxiety-like behavior (195). These represent core themes of current research. The keywords are divided into nine clusters, each represented by a different color. Cluster 1 (shown in red) mainly focuses on themes directly related to anxiety and depression, including “anxiety”, “anxiety disorders”, “depression”, and “major depression disorder”. Cluster 2 (green) is mainly related to basic experiments, including terms such as “mice”, “models”, and “mouse models”. Cluster 3 (dark blue) mainly involves pathophysiology, including terms such as “protein coupled receptor”, “indoleamine 2,3-dioxygenase”, and “vagus nerve stimulation”. Cluster 4 (yellow) is mainly related to neurological diseases, including “central nervous system”, “Alzheimer’s disease”, and “multiple sclerosis”. Cluster 5 (purple) mainly focuses on the risk factors for children and adolescents, including “children”, “early life”, and “brain development”. Cluster 6 (light blue) is mainly related to dietary behavior, including “nutrition”, “eating disorders “, and “food intake”. Cluster 7 (orange) is mainly related to the gut-brain axis of microorganisms, including “gut-brain axis”, “dynamics”, and “gastrointestinal microbiome”. Cluster 8 (brown) is mainly related to intestinal diseases, including “inflammatory bowel disease”, “ulcerative colitis”, “colitis”, etc. Cluster 9 (pink) is mainly related to probiotics and immune response, including “probiotics”, “immune function”, “bifidobacteria”, and “immune system”.

As shown in **Figure 6B**, We also analyzed the temporal changes in keyword co-occurrences and found that terms such as “sleep deprivation”, “prebiotic”, “tryptophan metabolism”, “gut- microbiota-brain axis”, and “internal flora” have frequently emerged in recent years. These keywords may provide new insights into the pathogenesis and treatment approaches for anxiety disorders.

### 3.6 Co-cited references and reference bursts

**Figure 7:**
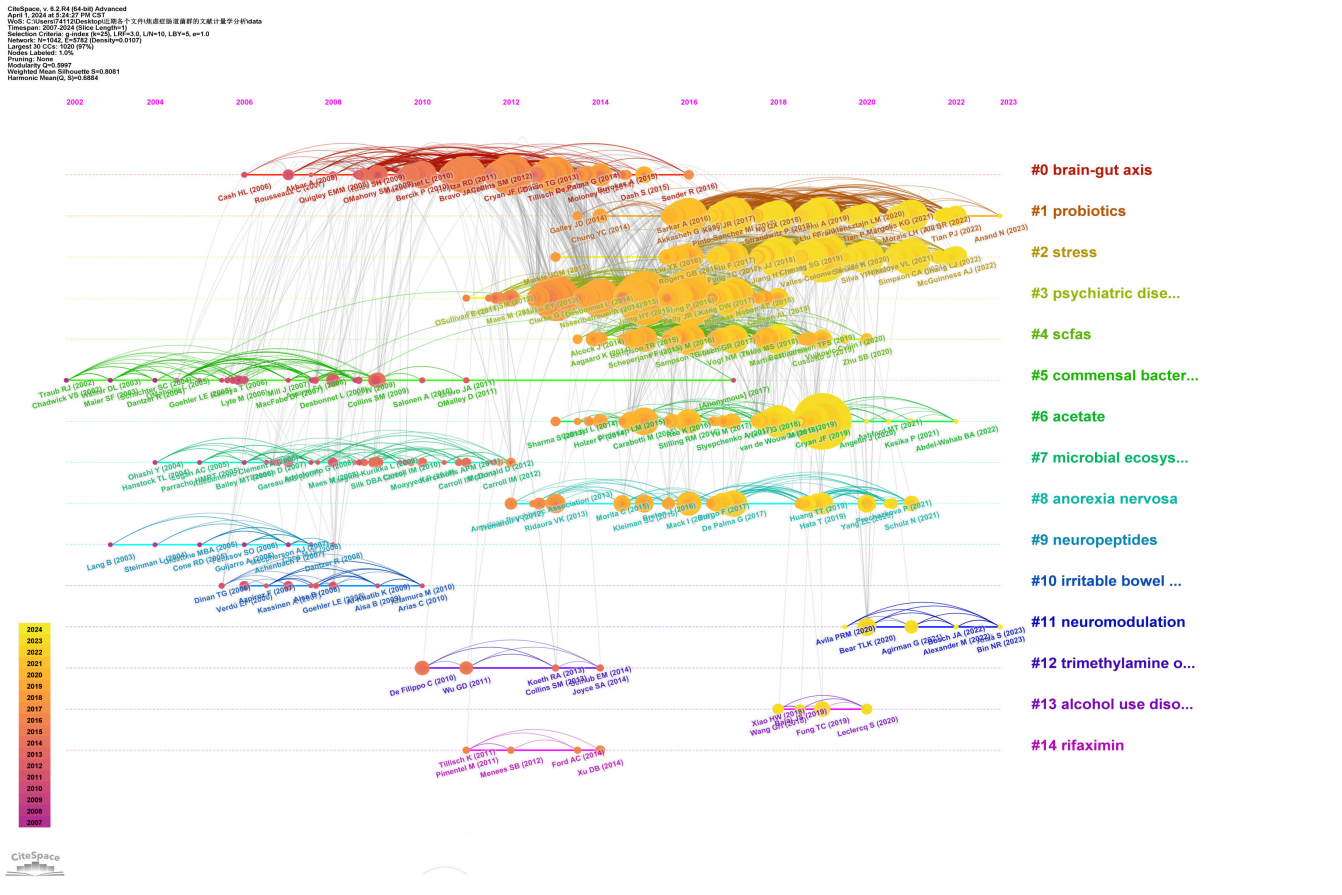
The timeline graph of the co-cited references.

Highly cited references often indicate influence in the research field, leading to new research directions. We used CiteSpace to visualize the top 25 most-cited studies on anxiety disorders and gut microbiota (**Table 4**). The timeline and citation frequency in the **Figure 7** show that larger circles represent higher citation frequencies. For example, John Cryan’s article “Mind-altering microorganisms: the impact of the gut microbiota on brain and behavior” published in Nature Reviews Neuroscience in 2012, had a citation burst intensity of 39.4429 and remained influential until 2017, demonstrating its profound impact in the field of microbial gut-brain axis research. Gerard Clarke’s 2013 article, “The microbiome-gut-brain axis during early life regulates the hippocampal serotonergic system in a sex-dependent manner” published in Molecular Psychiatry, had a burst intensity of 37.0662, with lasting influence from 2014 to 2018, indicating a long-lasting impact in this research field and providing a new perspective. Citation burst analysis shows that the most frequent citation bursts occurred between 2011 and 2016, indicating peak interest during that period and drawing significant attention from researchers.

**Table 4:** Top 25 references with the highest citation bursts related to anxiety disorder and gut microbiota.

Rank	Author	Journal	DOI	Strength
1	Cryan JF	Nat Rev Neurosci	10.1038/nrn3346	39.4429
2	Clarke G	Mol Psychiatr	10.1038/mp.2012.77	37.5808
3	Bravo JA	P Natl Acad Sci Usa	10.1073/pnas.1102999108	37.0662
4	Jiang HY	Brain Behav Immun	10.1016/j.bbi.2015.03.016	35.7781
5	Neufeld KM	Neurogastroent Motil	10.1111/j.1365-2982.2010.01620.x	35.4226
6	Heijtza RD	P Natl Acad Sci Usa	10.1073/pnas.1010529108	35.4226
7	Cryan JF	Physiol Rev	10.1152/physrev.00018.2018	31.265
8	Bercik P	Nat Rev Gastro Hepat	10.1053/j.gastro.2011.04.052	29.6052
9	Hsiao EY	Cell	10.1016/j.cell.2013.11.024	28.3053
10	Tillisch K	Nat Rev Gastro Hepat	10.1053/j.gastro.2013.02.043	27.5989
11	Messaoudi M	Brit J Nutr	10.1017/S0007114510004319	27.124
12	Kelly JR	J Psychiatr Res	10.1016/j.jpsychires.2016.07.019	26.645
13	Foster JA	Trends Neurosci	10.1016/j.tins.2013.01.005	25.9937
14	Naseribafrouei A	Neurogastroent Motil	10.1111/nmo.12378	24.7993
15	Simpson CA	Clin Psychol Rev	10.1016/j.cpr.2020.101943	24.37
16	Zheng P	Mol Psychiatr	10.1038/mp.2016.44	23.7947
17	Gareau MG	Gut	10.1136/gut.2009.202515	22.2565
18	Bercik P	Neurogastroent Motil	10.1111/j.1365-2982.2011.01796.x	22.1682
19	Collins SM	Nat Rev Microbiol	10.1038/nrmicro2876	21.4225
20	Dinan TG	Biol Psychiat	10.1016/j.biopsych.2013.05.001	21.3795
21	Bercik P	Nat Rev Gastro Hepat	10.1053/j.gastro.2010.06.063	21.1299
22	Morais LH	Nat Rev Microbiol	10.1038/s41579-020-00460-0	20.9182
23	Erny D	Nat Neurosci	10.1038/nn.4030	19.0383
24	Desbonnet L	Mol Psychiatr	10.1038/mp.2013.65	18.893
25	Bailey MT	Brain Behav Immun	10.1016/j.bbi.2010.10.023	18.2115

## 4 Discussion

### 4.1 General information

This study used VOSviewer and CiteSpace software for bibliometric analysis to explore publication trends, countries, institutions, journals, authors, keywords, and references of literature in the field of anxiety disorders and gut microbiota from 2004 to 2023. Over the past two decades, the number of studies on this topic has steadily increased, reflecting growing attention from scholars. Notably, China leads in the number of published articles, though the overall and average citation counts remain relatively low. This suggests that while Chinese researchers show strong interest in the field, the depth of their research may need further development. In contrast, Ireland not only ranks highly in publication volume but also exceeds in citation frequency, indicating significant contributions to this field. For example, John Cryan from University College Cork, Ireland, made a notable impact with his 2012 paper “Mind altering microorganisms: the impact of the gut microbiota on brain and behavior” published in Nature Reviews Neuroscience. The paper focuses on the mechanisms by which gut microbiota regulates physiological and psychological changes in the human body, suggesting that gut microbiota affects brain function via pathways involving the nervous, immune, and endocrine systems (15). Similarly, the 2019 article “The Microbiota-Gut-Brain Axis” highlights the relationship between gut microbiota and various neurological and psychiatric disorders. These studies are of great significance for understanding the relationship between gut microbiota and anxiety, offering new perspectives on the pathogenesis of anxiety disorders.

### 4.2 Research hotspots

Through keyword analysis, this study identifies the major research topics in the relationship between anxiety disorders and microbiota, including anxiety disorder symptoms, risk factors, comorbid mental and neurological disorders, underlying mechanisms, and treatments. This overview provides insight into the current global research landscape and highlights key areas for future focus.

#### 4.2.1 Anxiety disorders and their symptoms

Anxiety disorders often emerge during adolescence and early adulthood (16). In addition to feelings of nervousness and irritability, individuals often experience symptoms such as poor concentration, fatigue, sleep disorders, chronic headaches, and gastrointestinal issues (6). One example of a related gastrointestinal condition is irritable bowel syndrome (IBS), by intestinal dysfunction leading to symptoms such as abdominal pain, bloating, and changes in bowel habits (17). IBS is thought to result from interactions between gut microbiota and central nervous system dysfunction (18). Psychological factors, such as stress, may also influence IBS, possibly through mechanisms involving the gut-brain axis and intestinal permeability (19). Since IBS is commonly comorbid with anxiety, further research into the connection between these two conditions is needed, which could reveal new therapeutic targets for treating both IBS and anxiety.

#### 4.2.2 Risk factors

The risk factors for anxiety disorders in children and adolescents mainly include gender, age, place of residence, stress, parental history of anxiety, and childhood adversity. Generally, girls have a higher incidence of anxiety disorders than boys, and the likelihood of developing anxiety increases with age. Children and adolescents living in cities are more prone to anxiety disorders than those living in rural areas. Additionally, children whose mothers have anxiety disorders are more likely to develop the condition themselves. Childhood adversity, such as parental divorce or the loss of close relationships, also increases the risk (16, 20).

In adults, risk factors for anxiety disorders mainly include being female, having a low education level, low socio-economic status (20), substance use (such as alcohol and smoking), and avoidance behaviors (21). Understanding these risk factors is essential for enabling early intervention and management, which can reduce the national burden of anxiety-related diseases.

#### 4.2.3 Co-morbidity

Anxiety disorders frequently co-occur with various mental and physical illnesses, including attention deficit hyperactivity disorder (ADHD), depression, epilepsy, Parkinson’s disease, and cardiovascular disease (5). For instance, ADHD is categorized into emotional regulation disorders and concentration deficits, both of which overlap with anxiety symptoms (22). In childhood, anxiety can exacerbate inhibitory dysfunction in ADHD, while in adulthood, it may affect sleep quality in affected individuals (23). Severe depression also has a high comorbidity rate with anxiety disorders, ranging from 45.7 to 75.0% (24). When anxiety and severe depression are comorbid, the course of severe depression tends to be longer, with higher recurrence rates (25), increased suicide risk (26), and poor prognoses (27). Epilepsy has a similarly high morbidity with anxiety, likely due to shared pathological mechanisms such as neurotransmitter imbalances (e.g., GABA, norepinephrine) and changes in the limbic structures, especially the amygdala (28). Anxiety disorders and cardiovascular diseases share overlapping symptoms, making diagnosis challenging. They also share common pathophysiological mechanisms, especially in panic disorder and generalized anxiety disorder, where reduced heart rate variability indicates vagal dysfunction (29). The coexistence of anxiety disorders with other diseases can worsen patient outcomes, leading to lower treatment adherence and prolonged illness. Therefore, recognizing and addressing comorbid conditions in clinical practice is crucial, requiring further research into the mechanisms and treatments of anxiety disorder combined with other diseases.

#### 4.2.4 Gut microbiota

The human gastrointestinal tract is home to trillions of bacteria that play critical roles throughout the host’s life. Increasing evidence suggests that gut microbiota are key regulators of both gut and brain functions, not only aiding in digestion and vitamin synthesis but also influencing emotions and behavior (30). The gut microbiota undergoes changes with the host’s growth and development, yet it generally maintains a stable and diverse composition over time (31). A study of 7738 fecal samples from the Dutch microbiome project identified two significant genetic loci, namely near LCT and ABO, that influence gut microbiota composition. The study found that, in addition to environmental factors and diet, host genetics also affects the gut microbiota (32). The influence of gut microbiota on health largely stems from their metabolites, such as short-chain fatty acids (SCFAs), bile acids (BAs), tryptophan (Trp), and other metabolites, which can cause immune and inflammatory responses, potentially contributing to related diseases. These metabolites promote the function and differentiation of immunosuppressive cells such as tolerogenic macrophages (tMacs), regulatory T cells (Tregs), and tolerogenic dendritic cells (tDCs), thereby regulating the body’s immune response. Additionally, these metabolites regulate inflammation through receptor- mediated signaling pathways such as G-protein-coupled receptor 43(GPR43), aryl hydrocarbon receptor(AhR), and Takeda G-protein-coupled receptor 5(TGR5) (33).

#### 4.2.5 Mechanisms of anxiety disorders and gut microbiota

The gut-brain axis functions as a bidirectional system between the gut and the nervous system. The mechanisms involved include direct interactions, neurotransmitter regulation, immune responses, the endocrine system, and metabolic product effects (34).

##### 4.2.5.1 Direct action

A study by Javier A. Bravo, demonstrated that rhamnose supplementation in mice significantly reduced anxiety-like behavior. However, when the vagus nerve was severed, the effect disappeared, indicating that the vagus nerve is crucial for transmitting signals between the gut and the brain (35). The enteric nervous system, composed of intestinal wall neurons and glial cells, plays key roles in digestion and defense. It works closely with the central nervous system to maintain homeostasis (36). Neurons transmit electrical signals via action potentials, while oligodendrocytes support neurons by surrounding their cell bodies, synapses, and axons to ensure proper function (37). Glial cells also regulate synaptic transmission and maintain the BBB (38). Microglia, in particular, are essential for regulating neural circuit development (39). Research by Kabouridis suggests that the development and maintenance of glial cells are influenced by the gut microbiota (38). Further exploration of the relationship between glial cells and intestinal microbes could provide insights into the pathogenesis of anxiety disorders and inform the development of new treatment strategies.

##### 4.2.5.2 Neurotransmitter

###### 4.2.5.2.1 Gamma-aminobutyric acid

The amygdala plays an important role in regulating negative emotions in humans, and research has shown that anxiety disorders are strongly associated with persistent amygdala activation (40). GABA is a major inhibitory neurotransmitter in the central nervous system and is crucial in regulating amygdala activity and anxiety-related behaviors. GABA inhibitory effects on neurons are mediated by two receptor types: ionotropic GABAA receptors and metabotropic GABAB receptors. GABAA receptor features structural sites that regulate neuronal inhibition Common drugs such as barbiturates, benzodiazepines, and anesthetics, act on these sites to produce corresponding pharmacological effects (41, 42). GABAB receptors, which are G protein coupled, are widely distributed in the gastrointestinal tract and regulate intestinal motility, gastric emptying, and visceral pain perception (43). They reduce neuronal excitability by inhibiting adenylate cyclase, reducing Cyclic adenosine monophosphate (cAMP) levels, and regulating potassium and calcium ion channels. Increasing evidence suggests that gut microbiota such as Lactobacillus and Bifidobacterium can produce GABA, thereby influencing the gut-brain axis (44).

###### 4.2.5.2.2 5-hydroxytryptamine

Another important neurotransmitter, 5-HT, plays a key role in mental health. 5-HT is widely distributed in the brain, nerves, and gastrointestinal tract, where it regulates emotions, behavior, cognition, and gastrointestinal balance. Studies have identified seven different families and subgroups of 5-HT receptors, each affecting different brain regions and producing distinct effects. Receptors such as 5-HT1A, 5-HT1B, 5-HT1D, 5-HT2A, 5-HT2B, 5-HT2C, 5-HT3, 5-HT4, 5-HT6, and 5-HT7 are particularly associated with anxiety (45). Most 5-HT is synthesized and secreted by enterochromaffin (EC) cells. The gut microbiota plays a specific role in regulating the synthesis and secretion of 5-HT. For example, studies in germ-free mice have shown significantly lower levels of 5-HT in their colons and stools compared to specific pathogen-free mice (46). Recent evidence suggests that SCFAs, which are metabolites produced by the gut microbiota, promote the production and release of 5-HT by increasing the expression of tryptophan hydroxylase 1 (TPH 1) in colon endothelial cells (47).

###### 4.2.5.2.3 Dopamine

Dopamine, a monoamine neurotransmitter, plays a key role in regulating motor functions, motivation, reward, mood, and memory (48). It exerts its effects by binding to G protein-coupled receptors. The dopaminergic pathway involves neurons in the ventral tegmental area and the dense portion of the substantia nigra. These neurons project to the nucleus accumbens via the ventral tegmental area, the basal ganglia via the substantia nigra striata, and the limbic cortex via the midbrain’s limbic area. There is strong evidence linking gut microbes to the dopaminergic pathway. Studies indicate that gut microbes can modulate receptors in the dopaminergic pathway, protect dopamine neurons and slow dopamine depletion (49). For instance, dopamine levels in germ-free mice were significantly lower than in conventional mice (50). Additionally, *Lactobacillus casei* increases dopamine levels in the frontal brain of rats (51). However, the precise mechanisms underlying the interaction between gut microbes and dopamine remain unclear, necessitating further research to better understand this complex relationship.

###### 4.2.5.2.4 Noradrenaline

The noradrenergic system is mainly derived from the locus coeruleus and extends throughout the entire brain, limbic system, hypothalamus, cerebellum, and brainstem, enabling it to exert broad regulatory effects. Under stressful conditions, corticotropin-releasing factor activates the norepinephrine pathway, triggering norepinephrine release and inducing anxiety-like symptoms. In simple terms, overactivity of norepinephrine in the central nervous system causes anxiety-like symptoms (52). Certain microorganisms, such Mycobacteria, Escherichia coli, and Serratia marcescens microorganisms can produce 0.45 to 2.13 mM of norepinephrine *in vitro* (53). Furthermore, a study demonstrated that norepinephrine levels in the gut of germ-free mice are significantly lower than in conventional mice, and these levels can be restored through microbial colonization (54). While these findings suggest that gut microbes influence norepinephrine levels, the exact mechanisms remain elusive and require further investigation.

##### 4.2.5.3 Immunoreaction

A significant body of literature suggests that pro-inflammatory markers may be related to the occurrence of anxiety disorders. In a 1982 study, Henke et al. suggested that amygdala activity could have pro-inflammatory effects, while chronic inflammation may lead to heightened amygdala responses (55). This interaction between the amygdala and chronic inflammation may contribute to the occurrence and progression of anxiety disorders. Studies have found that patients with anxiety disorders tend to have elevated levels of CRP, lymphocyte count, and T cell count (56). The BBB functions not only as a selective exchange point of molecules and nutrients between the circulatory system and the brain but also as an immune barrier that helps maintain the homeostasis of the central nervous system by isolating peripheral inflammatory factors (57). Research by Braniste suggests that gut microbiota and its metabolites can affect the permeability of the BBB (58). When intestinal microorganisms are dysfunctional, the intestinal mucosal barrier is damaged and intestinal permeability is compromised, causing bacteria to release inflammatory factors in the circulatory system, increasing intestinal permeability. This allows bacteria to release inflammatory factors into the bloodstream, which activates peripheral immune cells, alters BBB permeability and allows inflammatory factors to enter the brain parenchyma, leading to anxiety-like behaviors (59).

##### 4.2.5.4 Hypothalamic-pituitary-adrenal axis

Stress is one of the most significant risk factors for anxiety disorders. It can activate the HPA axis, stimulating the synthesis and release of adrenocorticotropic hormone (ACTH), which in turn promotes the release of glucocorticoids such as cortisol (60). Excessive cortisol levels in the body can cause sustained tension and anxiety, increasing the likelihood of developing an anxiety disorder. The HPA axis and the gut-brain axis interact closely, leading to the activation of the HPA axis that can alter the structure and diversity of microorganisms (61), and to changes in gut microbiota which can affect the permeability of the intestinal barrier. This allows inflammatory factors, microbial antigens, and prostaglandins to penetrate the BBB and affect the activity of the HPA axis (62).

##### 4.2.5.5 Metabolic products

The gut microbiota produces hundreds of metabolites that play key roles in maintaining intestinal mucosal homeostasis, supporting immune system development, and providing energy. Here, we mainly discuss the effects of SCFAs, BAs, Trp on the gut-brain axis.

SCFAs, such as acetate, propionate, and butyrate, are byproducts of bacterial fermentation and help enhance the integrity of the BBB, thereby regulating its function. SCFAs also directly affect the activity of the enteric nervous system through the G protein-coupled receptor 41(GPR41), influencing the activity of the gut-brain axis (63). Additionally, SCFAs modulate the immune response by inhibiting histone deacetylase mRNA expression in macrophages (33).

BAs, through pathways involving the nuclear hormone receptor farnesoid X receptor (FXR) and the TGR5, play crucial roles in maintaining glucose metabolism, lipid metabolism, and immune response in the body. The activation of FXR affects intestinal mucosal permeability and reduces inflammatory cell production, while TGR5 activation inhibits lipopolysaccharide-induced inflammatory response (64). Further research is required to explore the relationship between gut microbiota, FXR, and TGR5.

Trp, the sole precursor of serotonin, is integral to the gut-brain axis. Some Trp crosses the BBB and is converted to 5-HT by tryptophan hydroxylase type 2, influencing emotions, cognition, and gastrointestinal balance (65). Alternatively, Trp can be broken down and metabolized into active canine uric acid, which is further broken down into kynurenic acid (KA) and quinolinic acid (QA) under the action of indoleamine 2,3-dioxygenase (IDO). These metabolites not only affect the central nervous system through the BBB but also mediate immune responses (66).

#### 4.2.6 Therapeutic method

Common treatment options for anxiety disorders include psychotherapy and medication. Cognitive-behavioral therapy (CBT) is the primary form of psychotherapy and is usually considered as the first-line treatment. Drug therapy is divided into two main categories. The first category includes antidepressants such as selective 5-HT reuptake inhibitors (SSRIs) and selective 5-HT-norepinephrine reuptake inhibitors (SNRIs), which are typically used as first-line medications. The second category consists of benzodiazepines, which are considered second- and third-line treatments for anxiety disorders (67). Given the gut microbiota’s influence on host cognition, emotions, and behavior, dietary interventions, probiotics, prebiotics, and fecal microbiota transplantation (FMT) for the treatment of anxiety disorders are receiving increasing attention.

##### 4.2.6.1 Probiotics

Probiotics are live microorganisms that provide health benefits to the host. They play a crucial role in maintaining intestinal integrity, promoting intestinal mucosal and BBB function, and enhancing neuronal survival and differentiation (68). Consuming probiotics can improve the composition of gut microbiota. As early as 2004, a study first demonstrated that yogurt fermented with Lactobacillus casei DN-114001 helped reduce stress-induced immune responses (69). Research by Professor Bravo et al. found that Lactobacillus rhamnosus (JB-1) significantly aids in treating anxiety disorders by regulating the neurotransmitter GABA (35). A study by Ruizhe Zhu randomly divided 60 anxious college students into two groups: one receiving a placebo and the other receiving the JYLP-326. After three weeks, the college students who received the probiotic JYLP-326 showed significant improvements in anxiety and insomnia symptoms (70). In a study focused on improving cognitive and emotional well-being in elderly individuals, probiotics containing Bifidobacterium BGN4 and Bifidobacterium longum BORI significantly reduced stress scores compared to a placebo (71). Nevertheless, a study by Professor Maria Ines Pinto-Sanchez revealed that while probiotics slightly reduced depression scores in patients with IBS, they did not show a significant impact on anxiety levels (72). Currently, there is insufficient empirical evidence to support the efficacy of probiotics in alleviating symptoms among individuals with anxiety disorders. Therefore, further investigations into the intricate interplay between the microbial-gut-brain axis and its impact on human physiology necessitate attention. While microbiotherapy may hold potential for treating anxiety disorders, its success is not guaranteed.

##### 4.2.6.2 Prebiotics

Prebiotics are chemical compounds that stimulate gut microbiota to produce beneficial effects on the host. The anti-anxiety effects of prebiotics are thought to be related to the increase of bifidobacteria in the intestine. In a pioneering study on prebiotics for treating IBS patients, those with anxiety showed a significant reduction in anxiety scores following oral administration of a specific dose of prebiotics (73). A study by Schmidt K et al. found that among 45 healthy individuals who took prebiotics daily, there was a significant reduction in salivary cortisol arousal responses, which influenced their mood (74).

Looking to the future, the use of prebiotics and probiotics for treating anxiety disorders is a promising field. Researchers should deepen their understanding of the specific mechanisms by which prebiotics and probiotics regulate gut microbiota and affect the central nervous system. Additionally, further exploration of the potential applications of prebiotics and probiotics in treating mental illnesses is essential. This could lead to the discovery of new therapeutic targets for more effective treatments of anxiety disorders in clinical practice.

##### 4.2.6.3 Dietary intervention

Nutritional psychiatry is an emerging field of research, with growing evidence supporting the positive effects of dietary in preventing and treating mental health disorders (75). Increasing research suggests that changes in dietary patterns can influence the onset and progression of anxiety disorders. Prominent examples include the Mediterranean diet, anti-inflammatory diets, and diets rich in diverse nutrients (75). A study on non-human female primates found that the western diet led to reduced gut microbial diversity and heightened activity in the sympathetic nervous system and hypothalamic-pituitary-adrenal axis compared to the Mediterranean diet group. Conversely, the Mediterranean diet reduced inflammatory responses and alleviated anxiety-like behaviors, demonstrating health benefits (76). Similarly, a cross-sectional study of older adults in Australia showed that adherence to a Mediterranean diet was inversely associated with anxiety symptoms (77). A meta-analysis showed that pro-inflammatory diets increase the risk of anxiety disorders by 66% compared to anti-inflammatory diets (78). Additionally, an analysis of the Dietary Inflammatory Index (DII) and mental health in adolescent girls found a significant negative correlation between DII scores and levels of anxiety and stress (79). Numerous studies also suggest that nutrients such as proteins, vitamins, minerals, carbohydrates, fats, and omega-3 fatty acids play a role in managing anxiety disorders (75). While these findings indicate that dietary patterns significantly impact mental health and suggest that changing dietary habits may be an effective intervention, more prospective studies are needed to establish a clear relationship between anxiety disorders and idiosyncratic diets.

##### 4.2.6.4 Fecal microbiota transplantation

FMT involves transferring feces from a healthy donor into the recipient’s gut to directly alter the recipient’s gut microbiota (80). Since its approval by the US Food and Drug Administration in 2013 for treating recurrent and refractory Clostridium difficile infections, FMT has garnered attention as a promising treatment for not only gastrointestinal disorders but also neurological, psychiatric, and metabolic disorders. A study on children with autism demonstrated significant changes in microbiota diversity following FMT, accompanied by improvements in autism-related behaviors (81). Another study found that transplanting gut microbiota from depressed patients into rats with depleted gut microbiota induced anxiety like behavior (82). Additionally, research on IBS patients with anxiety showed that FMT reduced anxiety scores and IBS symptoms (83). Despite these promising findings, the use of FMT for treating anxiety disorders requires further exploration. Its precise mechanisms remain unclear, though it is speculated that FMT may influence human emotions and cognition by regulating neurotransmitters and regulating the gut-brain axis. Whether FMT will become a mainstream treatment for anxiety disorders in the future remains uncertain and warrants additional research.

There are some limitations to our study. First, we solely relied on the Web of Science database for our literature analysis. As a result, we only analyzed articles and reviews, excluding other databases, which may have led to the omission of relevant literature. Second, our search was restricted to titles only. While this improved accuracy it may have caused us to miss some pertinent articles. Lastly, our search was limited to English-language literature, which could introduce potential bias. Current studies indicate that levels of dopamine, 5-HT, and norepinephrine are lower in germ-free mice compared to conventional mice (46, 50, 54). Additionally, the Mediterranean diet has been shown to reduce anxiety-like behavior in non-human female primates (76), while FMT can induce anxiety-like behavior in mice (82). However, findings are primarily based on animal studies, with limited data related to clinical trials. Future research should prioritize prospective cohort studies and clinical trials to validate these observations and advance their clinical applicability.

## 5 Conclusions

Our bibliometric analysis of gut microbiota research in the field of anxiety disorders shows a steady increase in publications since 2014. This area of research has attracted global attention, but greater collaboration among researchers from different countries could make the field more diverse and in-depth. Current research focuses include gut microbiota, gut-brain axis, pathogenesis, inflammatory response, and probiotics. Studying the role of gut microbiota in mental health has significant potential, and microbial therapy may become a prominent area of research for assisting in the treatment of anxiety disorders.
